# Identifying the Stressors Impacting Rescued Avian Wildlife

**DOI:** 10.3390/ani10091500

**Published:** 2020-08-25

**Authors:** Kimberley Janssen, Crystal Marsland, Michelle Orietta Barreto, Renae Charalambous, Edward Narayan

**Affiliations:** 1 School of Science and Health, Western Sydney University, Locked Bag 1797, Penrith, NSW 2751, Australia; 17724337@student.westernsydney.edu.au (K.J.); 18660193@student.westernsydney.edu.au (C.M.); r.charalambous@uq.net.au (R.C.); 2 School of Veterinary Sciences, Faculty of Science, University of Queensland, St Lucia, QLD 4072, Australia; m.barreto@uq.net.au; 3 School of Agriculture and Food Sciences, Faculty of Science, University of Queensland, St Lucia, QLD 4072, Australia; 4 Queensland Alliance for Agriculture and Food Innovation, University of Queensland, St Lucia, QLD 4072, Australia

**Keywords:** wildlife, environmental stress, urbanisation, birds

## Abstract

**Simple Summary:**

Stress evaluation in wildlife is valuable tool for rehabilitation and injury prevention. This pilot study investigated categories of stress in rescued birds. We determined three categories of stressors (preliminary, primary and secondary) using clinical data of rescued birds from Adelaide, South Australia. It was discovered that birds are highly susceptible to impact injuries (e.g., flying into a building window) and vehicle-related injuries as preliminary stressors, which often result in hospitalisation of birds. Immobility and abnormal behaviour represented the most common primary stressor, while the most common secondary stressors included trauma and fracture. Furthermore, the most common outcome in clinics due to exposure of birds to these three stressor categories was euthanasia.

**Abstract:**

Urbanisation exposes avian wildlife to an array of environmental stressors that result in clinical admission and hospitalisation. The aim of this pilot study was to conduct a retrospective analysis of clinical data and characterise this based on categories of stress experienced by avian wildlife patients. The results from this study indicated that impact injuries (*n* = 33, 25%) and vehicle-related injuries (*n* = 33, 25%) were the most common occurring preliminary stressors that resulted in the hospitalisation of avian wildlife. The most common outcome of avian patients that suffered from vehicle-related injuries was euthanasia (*n* = 15, 45%), as was avian patients that suffered from impact injuries (*n* = 16, 48%). Immobility (*n* = 105, 61%) and abnormal behaviour (*n* = 24, 14%) were the most commonly occurring primary stressors of avian patients. Finally, trauma (*n* = 51, 32%) and fractures (*n* = 44, 27%) were the most common occurring secondary stressors in avian patients. The most common outcome of all these stressors was euthanasia. This study provided further evidence towards the notion that human- and urbanisation-related stressors are the main causes of hospitalisation of avian wildlife, but also indicated that birds admitted as a result of human-related stressors are more likely to be euthanised than released. This study also provided a categorisation system for the stressors identified in avian wildlife patients (preliminary, primary and secondary) that may be used to monitor the stress categories of wildlife patients and gain a deeper understanding of the complex notion of stress.

## 1. Introduction

Clinical treatment for injured avian wildlife is well explored within the literature [1,2,3], however, there is limited information regarding the long-term impacts that environmental stress has on the recovery of a patient. Environmental stressors are factors within the environment that cause stress to an individual [4]. Examples of environmental stressors include biotic factors, such as limited/reduced food availability, presence of predators, existence of pathogenic organisms, and interactions with conspecifics [4]. Alternatively, abiotic factors exist, such as extreme temperatures, reduced water availability, and the presence of toxicants [4]. Currently, the main limitation in clinical avian care research is that little is known about how environmental stressors affect avian wildlife.

The universal meaning of stress has been difficult to define. Moberg [5] defined stress as ‘the biological response elicited when an individual perceives a threat to its homeostasis’. This definition has since been debated particularly due to the word “homeostasis” [6]. Nevertheless, it is generally agreed upon that stress is a biological response, termed as the stress response, that occurs when an animal is presented with an unpleasant stimulus known as a stressor [7,8]. Stress is not inherently harmful; however, ongoing stress has pervasive consequences for the well-being of animals and dictates the long-term survival and quality of life of veterinary patients [9,10,11]. When animals encounter environmental stressors, the hypothalamic−pituitary−adrenal (HPA) axis is activated, which prepares the body for some form of exertion [12,13]. The hypothalamus then releases a hormone called corticotrophin releasing factor (CRF), which signals the anterior pituitary to release a hormone called adrenocorticotrophic hormone (ACTH) [12,13]. Adrenocorticotrophic hormone circulates in the blood and results in an increased output of glucocorticoids from the adrenal cortices [12,13]. Glucocorticoids act to divert the storage of glucose as glycogen, and to instead mobilise glucose from stored glycogen [12,13]. The most pivotal glucocorticoid within the HPA axis is cortisol, and it works to stimulate gluconeogenesis [12,13]. Gluconeogenesis acts in a way that prepares the animal for a physical challenge by partitioning energy and also acts as a chemical blocker within the negative feedback process [12,13]. Since the HPA axis comes at a cost of diverting energy away from corporal bodily functions, long-term exposure to environmental stressors can reduce growth, reproduction, and immune function in animals [12].

Four categories have been used to quantify stress in fish [9]. These categories include primary stress, secondary stress, and tertiary stress [9]. For the purpose of this study, these categories were adapted to avian patients in clinical care and a fourth category, preliminary stress, was introduced. Preliminary stress refers to the initial causative factor that resulted in a patient requiring any sort of treatment in a clinical setting. A preliminary stressor is anything that can cause any physical or psychological stress to an individual. This may include an animal attack, vehicle collision or heat stress. Primary stress refers to the effect caused by preliminary stress including any physical or behavioural abnormalities [9]. This may include abnormal behaviour, feather damage or bleeding. Secondary stress refers to the diagnosis which resulted in or caused the preliminary stressor [9]. This may include fractures, disease and infection. Tertiary stress refers to a long-term stressor that may impact a patient after the other stressors have been treated [9]. This may include brain damage, permanent body disfigurement and loss of sight or other senses. For example, if a bird flew into a window, it would have experienced a preliminary stressor. If the wings of this bird had begun to bleed, it would have experienced the bleeding as a primary stressor. If this bleeding was due to a broken bone which had punctured the skin, it would have experienced the fracture as a secondary stressor. Finally, if the broken bone had resulted in permanent body disfigurement and an inability to fly properly, it would have had experienced a tertiary stressor. Beyond categorising the complex notion of stress for the purpose of gaining a clearer understanding of this biological phenomenon, this would also help us to minimise the intensity and frequency of stress experienced by animals, which are two very significant characteristics of stress involved in wildlife recovery [10,11,12,13]. Therefore, it is integral to quantify the chain of stressors experienced by wildlife in clinical care for the control of these stressors from when the bird is rescued, throughout treatment and after release or rehoming.

Current clinical data surrounding the assessment and management of stress in avian wildlife admitted to clinical care are often difficult to follow. This is due to stress management often requiring invasive methods such as blood collection. However, research using existing records from wildlife hospitals could be used as a tool to better understand avian preservation efforts, particularly of species under conservation [14,15,16,17,18,19]. Furthermore, these databases can help us understand the impact of human activities on wildlife in a particular geographic location and how this impact varies among different avian species, age and human rural versus urban living environments [19]. Lastly, wildlife records could also illuminate the typical outcome of avian recues i.e., the likelihood of recovery and release versus death, and the circumstances surrounding these outcomes.

The aim of this study was to conduct a retrospective analysis of clinical data and characterise this based on categories of stress experienced by avian wildlife patients admitted to a wildlife clinic. This form of clinical intervention aims to serve as a database for ecological research and urban planning.

## 2. Materials and Methods

This study was conducted in collaboration with the Adelaide Koala and Wildlife Hospital (AKWH), located in Plympton, South Australia. Clinical data for avian wildlife patients presented to the hospital between 2014 and 2017 were collected on site at the AKWH. The clinical data collected were used to obtain information on the stressors experienced by avian wildlife patients throughout their stay at the AKWH. These data were then systematically collaborated in a Microsoft Excel document and classified according to the patient’s age (egg, nestling, juvenile, or adult), species (magpie, lorikeet, ibis, kookaburra etc.), their classification of stress (preliminary, primary, secondary), and finally, the outcome of that diagnosis (euthanasia, care, release etc.). Tertiary stress unfortunately was not able to be investigated to the expected extent and was intended based on the long-term outcome in correlation to the severity of the patient’s condition due to lack of clinical records. Therefore, this category was omitted.

The location in which the birds were found was categorised based on a method outlined by Narayan and Vanderneut [20] and criteria provided by the Australian Bureau of Statistics. Locations were provided by suburb and we used Google maps and location demographics to categorise the suburbs as urban, rural or rural−urban. A location was categorised as “urban” if it was densely population and included a population of more than 1000 people. A location was categorised as “rural” if it included was sparsely populated and consisted of mainly open land and contained few buildings. Finally, an area was described as rural−urban if it was situated near or on a fringe between rural and urban areas and if it was populated to a lesser extent than urban areas but more so than rural areas.

An important caveat to note here is that the data provided were not always comprehensive and there were some information gaps. For example, all entries from 2015 were missing and unable to be collected, and some of the provided entries were missing some information, such as the bird’s species or location found. For this reason, the data were too unstable to complete statistical analysis beyond the scope of a descriptive analysis. The purpose of this preliminary study, however, was not to analyse the data per year but to create an average to be used for discussion purposes.

## 3. Results

A total of 178 records pertaining to birds rescued in 2013 (*n* = 6), 2014 (*n* = 37), 2016 (*n* = 51) and 2017 (*n* = 84) were collected (Supplementary Data: Table S1). The majority of birds were rescued from urban areas (*n* = 135), followed by rural (*n* = 6) and rural−urban (*n* = 6) areas. Note that 31 birds were missing location information. The total number of records was comprised of 25 different types of bird. Of these, lorikeets were the most commonly rescued (*n* = 46), followed by magpies (*n* = 25) and cockatoos (*n* = 23). The most common age group of rescued birds was adult (*n* = 143), followed by juvenile (*n* = 20) and nestling (*n* = 15).

**Figure 1:**
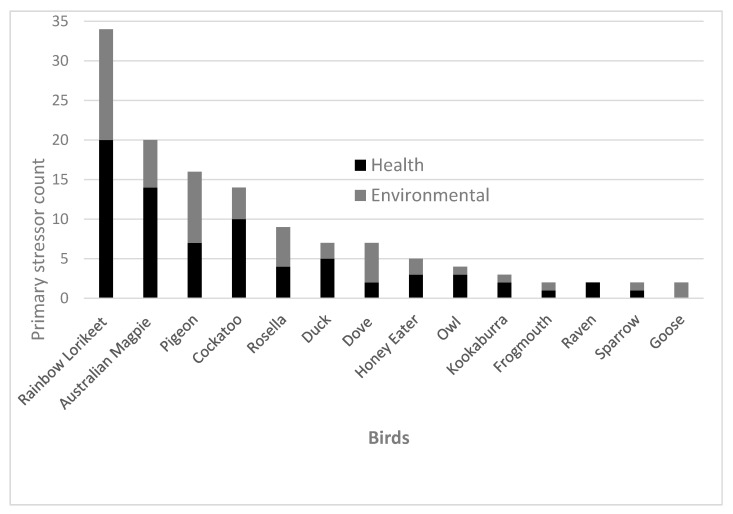
Key preliminary stressors experienced by majority of the avian patients admitted to the Adelaide Koala and Wildlife Hospital in 2013, 2014, 2016 and 2017 (*n* = 138). Preliminary stressors were pooled into two categories (health or environmental). Health-related preliminary stressors comprised of factors such as impact injury, vehicle trauma, fallen onto ground from substrate, lice presence, severely wet (unable to fly) and genetic issue (not known). Environmental-related preliminary stressors included factors such as animal attack, abnormal behaviour, bullied, rubbish attached, abandoned, ant attack and heat stress. Refer to Supplementary Table S1 to access raw data related to all bird patients, as not all birds have been shown in the above graph where the preliminary stressor count was only one per bird species.

Results from this study show that impact injuries (*n* = 33, 25%) and vehicle-related injuries (*n* = 33, 25%) were the most common occurring preliminary stressors which caused hospitalisation of avian patients (Figure 1). Note that vehicle injuries may be documented as impact injuries if there were no witnesses or evidence that a car was involved upon the admission of a bird. The most common outcome of avian patients that suffered from vehicle injuries was euthanasia (*n* = 15, 45%) and only 18% (*n* = 6) were released back into their ecosystem. Likewise, the most common outcome for avian patients that had suffered from impact injuries was also euthanasia (*n* = 16, 48%). A previous study reported that impact injuries are not typically fatal events for birds [21]. However, our results indicate that only 27% (*n* = 9) of avian patients admitted due to impact injuries were able to be released back into their ecosystem. The remaining patients had no outcome information, died due to their injuries or were kept in care and no further information was provided.

As mentioned in the above caption for Figure 1, abnormal behaviour referred to behaviours that are abnormal for that bird species and age group that were not characterised as immobility. Animal attack (*n* = 4) also included cat (*n* = 15) and dog attacks (*n* = 5). Impact injury included any trauma from events such as flying into a window or building which was not related to vehicle collisions and which did not fit any of the other categories. Abandoned refers to a young bird which was separated from its mother and found alone. Likewise, fallen from nest refers to a young bird who fell but did not land in a pool of water. In contrast, fell in pool could refer to a bird of any age that fell in a pool of water. Bullied refers to birds which had experienced bullying behaviour from other more dominant birds to the point where they required clinical care. Wet included birds which had experienced difficulty flying or locomoting due to wet weather conditions.

Ecological groupings of bird species within preliminary stressors (Figure 1) were as follows: Cockatoo (*n* = 21) also included yellow-tailed black cockatoo (*n* = 1), sulphur-crested cockatoo (*n* = 2) and galah (*n* = 9). Magpie (*n* = 19) included one Murray magpie (*n* = 1). Honey eater (*n* = 1) also included noisy miner (*n* = 2), native miner (*n* = 1) and wattle bird (*n* = 1). Dove (*n* = 6) also included one spotted dove (*n* = 1). Lorikeet (*n* = 31) also included one musk lorikeet (*n* = 1). Owl (*n* = 1) also included boobook owls (*n* = 3). There were some birds presented to the hospital whose preliminary stressor was unable to be identified (*n* = 40) and thus were omitted from this figure.

**Figure 2:**
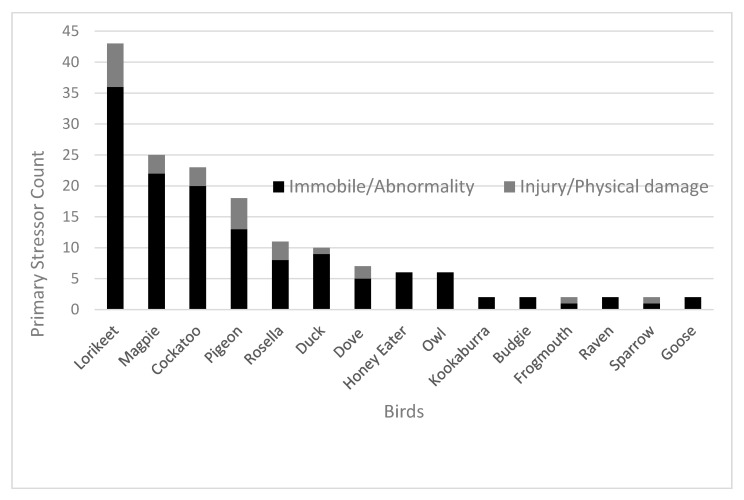
Key primary stressors experienced by the majority of avian patients admitted to the Adelaide Koala and Wildlife Hospital in 2013, 2014, 2016 and 2017 (*n* = 173). Note that birds presented to the hospital whose primary stressor was unable to be identified (*n* = 5) or those species with a cumulative count of only one primary stressor were omitted from this figure. Primary stressors were pooled into two categories. Immobile/abnormality-related primary stressors comprised of factors such as physical abnormality, abnormal behaviour and immobile. Injury/physical damage-related primary stressors included factors such as dislocation, oil, damaged feet, diarrhoea, superficial injury, feather damage and bleeding. Refer to Supplementary Table S1 to access raw data related to all bird patients.

Primary stress refers to the effect caused by preliminary stress including any physical or behavioural abnormalities. Immobility (*n* = 105, 61%) and abnormal behaviour (*n* = 24, 14%) were the most common occurring primary stressors (Figure 2). The most common outcome of avian patients that suffered from both immobility and abnormal behaviour was euthanasia at 50% (*n* = 52) and 38% (*n* = 9), respectively.

Within the primary stressor category (Figure 2), cockatoo (*n* = 3) also included yellow-tailed black cockatoo (*n* = 1), sulphur-crested cockatoo (*n* = 2), galah (*n* = 15) and corella (*n* = 2). Magpie (*n* = 24) also included one Murray magpie (*n* = 1). Honey eater (*n* = 2) also included noisy miner (*n* = 2), native miner (*n* = 1) and wattle bird (*n* = 1). Dove (*n* = 6) also included one spotted dove (*n* = 1). Lorikeet (*n* = 42) also included one musk lorikeet (*n* = 1). Owl (*n* = 2) also included boobook owl (*n* = 4).

**Figure 3:**
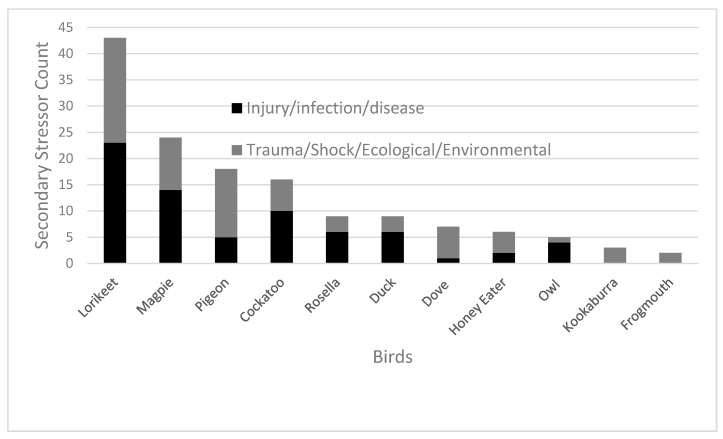
Key secondary stressors experienced by avian patients admitted to the Adelaide Koala and Wildlife Hospital in 2013, 2014, 2016 and 2017 (*n* = 161). Note that birds presented to the hospital whose secondary stressor was unable to be identified (*n* = 17) or those species with a cumulative count of only one secondary stressor were omitted from this figure. Secondary stressors were pooled into two categories. Injury/infection/disease-related secondary stressors comprised of factors such as fractures, dislocation, feather damage, broken bone etc. Trauma/shock/ecological/environmental-related primary stressors included factors such as severe dehydration, shock, shot, tissue damage from heat stress etc. Refer to Supplementary Table S1 to access raw data related to all bird patients.

Secondary stress refers to the diagnosis underlying that of which resulted in or caused the preliminary stressor. Trauma (*n* = 51, 32%) and fractures (*n* = 44, 27%) were the most common occurring secondary stressors (Figure 3). The most common outcome of avian patients that suffered from trauma was euthanasia for both trauma (*n* = 18, 35%) and fractures (*n* = 25, 57%).

Within the secondary stressor data shown in Figure 3, the ecological groupings were as follows: Cockatoo (*n* = 2) also included yellow-tailed black cockatoo (*n* = 1), sulphur-crested cockatoo (*n* = 2), galah (*n* = 10) and corella (*n* = 1). Magpie (*n* = 23) also included one Murray magpie (*n* = 1). Honey eater (*n* = 2) also included noisy miner (*n* = 2), native miner (*n* = 1) and wattle bird (*n* = 1). Dove (*n* = 6) also included one spotted dove (*n* = 1). Lorikeet (*n* = 42) also included one musk lorikeet (*n* = 1). Owl (*n* = 2) also included boobook owl (*n* = 3).

## 4. Discussion

The results from this study, although restricted to South Australia, can be interpreted on a broader context. Avian ecosystems have undergone profound change due to the increasing threat of urbanisation, which creates disparity in the richness and diversity of the environment [22,23,24]. Urbanisation challenges avian species by creating threats to their survival through decreased food availability and increased air, light and noise pollution, which results in compromised immune function due to stress [25]. This study allowed these impacts to be quantified by investigating avian patients and, in doing so, identified the risks faced by avian wildlife and the mortality which results from these. Previous studies have attested that anthropomorphically sourced stressors are the main challenges affecting avian wildlife vitality [26,27,28], and this is consistent with the results of this study. In fact, the preliminary stressors of impact injury, vehicle and rubbish attached, which are all anthropomorphically sourced stressors, accounted for 53% of hospitalisations and 47% of total deaths over a four-year period. These results also indicated that birds which suffer from these stressors are less likely to recover. Furthermore, this study highlighted the fact that these stressors linked to human behaviour are impacting a wide range of avian species. This is important as although the vast majority of the birds identified were not vulnerable or endangered species, the number of wildlife species at risk of endangerment are continuously increasing due to the direct effects of urbanisation and human-related stressors [29]. This indicates that there is much work to be done in order to better preserve Australian avian wildlife.

After implementing a categorical method of assessing stress, veterinary clinics will be able to establish and address different avian stressors, and hopefully implement practices to avoid further hospitalisation and improve mortality rates. When birds suffer with broken bones, their chance of survival is dramatically decreased [14]. This may be due to reasons beyond the severity of the stress experienced by the bird such as the difficulty associated with casting and splinting bones on small mammals [14], and this may also be the reason behind the high euthanasia outcome seen in this study. Furthermore, although it was not able to be investigated in this study, exposure to broken bones may lead to long-term suffering and discomfort from tertiary stressors [12]. These long-term impacts could be due to permanent disfiguration, which can lead to a compromised ability to fly and survive [20]. In our case, the clinic contained no data on the survival rates of an individual or if the patient was ever re-admitted to the same or another clinic. It is recommended that long-term monitoring of once admitted avian patients could be performed after they are released into their natural ecosystems. This would provide statistical data on the tertiary stressors of avian wildlife post-rehabilitation, being that long-term stressors impact a patient after the underlying stressors have been treated. In order to be unnecessarily exhaustive with resources, this long-term monitoring could be used on a selection of patients, such as endangered, native species.

It is important to make note that not all possible clinical cases resulted in rehabilitation, in particular, our results showed that avian patients that were received with bone fracture (*n* = 25, 57%) were euthanised. The plausible reason for this outcome is due to the length of care and further interaction with human carers that may be required for wild birds that have undergone successful clinical surgery such as bone fracture repair. This could be difficult to manage, especially because wild birds may not be easily desensitised to frequent human exposure, and also because some species of birds (such as rare black cockatoo) may be rehabilitated with a wildlife carer more easily than more commonly occurring species (e.g., magpie). Therefore, a combination of human resource issues as well as infrastructure resource issues may limit the clinical intervention of specific clinical cases such as a bone fracture of wild avian patients, although veterinarians are well trained to perform bone fracture surgeries in avian patients.

Previous studies have highlighted that responses to urbanisation may be species specific. For example, some species of birds disappear completely from an area once the area becomes urbanised, and other species remain and dominate [30]. The categories of stress established in this study can be used to identify species-specific trends of stress, that is, how different species vary in their susceptibility to certain stressors. However, this would involve a level of standardisation for findings to be consistent and reliant. It would be advantageous if everybody (from foster careers to veterinarians) could assess stress using the same formula of evaluation so as to prevent potential disparities. Finally, wildlife recues have been termed potential sentinels of ecosystem health [31]. Although this study was limited in its lack of specifically when it came to diseased patients or those with a microbial or parasitic infection, this is an area for future research which would be particularly useful.

## 5. Conclusions

This study has contributed fundamental research towards understanding the different categories of stress experienced by avian patients requiring clinical treatment. By organising stressors as preliminary, primary or secondary, this allowed for a clearer understanding of the chain reaction between environmental stress and avian wildlife. Furthermore, this study demonstrated that human- and urbanisation-related stressors were the most common stressors which lead to the hospitalisation and death of birds over a four-year period. In the future, it would be advantageous to monitor tertiary stress in order to allow for an evaluation of birds’ wellbeing in the long term. Similarly, these categories of stress could be used to identify species-specific trends and identify which species cope more effectively with urbanisation and which species are more at risk of dying out due to human activity.

## 6. Patents

Ethics approval and consent to participate: Animal Care and Ethics Committee (ACEC) of the Western Sydney University approved this retrospective study (Approval number: A12145). And we obtained consent from the Adelaide Koala and Wildlife Hospital to publish our research.

## Data Availability

Clinical data on individual patients are held by the Adelaide Koala and Wildlife Hospital database which is not available to the public. Data relevant to this study are presented in the Results section.

## Supplementary Materials

The following are available online at https://www.mdpi.com/2076-2615/10/9/1500/s1, Table S1: Raw Data Related to Bird Patients.
